# Lens Biometry in Congenital Lens Deformities: A Swept-Source Anterior Segment OCT Analysis

**DOI:** 10.3389/fmed.2021.774640

**Published:** 2021-12-20

**Authors:** Ze-xu Chen, Wan-Nan Jia, Yong-Xiang Jiang

**Affiliations:** ^1^Eye Institute and Department of Ophthalmology, Eye & ENT Hospital, Fudan University, Shanghai, China; ^2^NHC Key Laboratory of Myopia (Fudan University), Key Laboratory of Myopia, Chinese Academy of Medical Sciences, Shanghai, China; ^3^Shanghai Key Laboratory of Visual Impairment and Restoration, Shanghai, China

**Keywords:** microspherophakia, coloboma lentis, posterior lenticonus, CASIA2, lens biometry

## Abstract

**Aims:** To investigate the lens biometric parameters in congenital lens deformities, using a novel technique of swept-source anterior segment optical coherence tomography (SS-ASOCT).

**Methods:** This prospective study included patients with microspherophakia (MSP), coloboma lentis (CL), and posterior lenticonus (PL). For this cohort, 360-degree high-resolution lens images were obtained using the latest SS-ASOCT (CASIA2, Tomey Corp, Nagoya, Japan). The lens biometric parameters were calculated by the CASIA2 built-in software for anterior lens radius (ALR), posterior lens radius (PLR), anteroposterior distance (APD), anterior chamber depth (ACD), equatorial diameter (Eq Dia), rear projection length (RPL), and maximum diameter of the lesion (MDL).

**Results:** This study included two eyes each with MSP and CL and one eye with PL. The lens of MSP was spherical and posteriorly dislocated, with decreased ALR and PLR, Eq Dia, but increased APD. In patients with CL, the coloboma was isolated, bilateral, inferior, and located toward the maldeveloped ciliary body. High astigmatism was mainly lenticular, and this was calculated by the ALR and PLR. Regarding the site of coloboma, a significant decrease in ALR was observed, while the PLR and APD were not affected. The PL eyes had a cone-shaped protrusion of the posterior lens surface with a subtle cataractous region around the apex. An extremely high posterior surface curvature was observed with a mean PLR of 1.67 mm. The RPL and MDL were about 1.80 and 0.4 mm, respectively, which were homogenous at different sections.

**Conclusions:** The CASIA2 is a valuable option for *in vivo* crystalline lens measurement for congenital lens deformities, enabling the accurate diagnosis and providing illuminating insights into the pathogenesis of MSP, CL, and PL

## Introduction

The crystalline lens originates from the surface ectoderm overlying the optic vesicle from the 4th week of intrauterine life (1). However, any abnormal condition during embryonic development may lead to the occurrence of the congenital lens deformity, which can result in the anomaly of the crystalline lens shape since or before birth (2). The lens form is modulated by the strength arising from the zonular apparatus due to the applied or released load near the lens equator (3). In microspherophakia (MSP), the growth of the secondary lens fibers gets arrested as the zonules are extensively compromised (4). In contrast, the coloboma lentis (CL) is the notch of lens tissue at the lens equator, caused by the segmental loss of zonules (5). Moreover, the tension of the capsule also contributes to the lens shape (6). The regional weakness of the posterior capsule and subsequent herniation of the cortical lens fibers are the pathogenesis of posterior lenticonus (PL) (7, 8). Due to their scarcity, the progress, diagnosis, and treatment of these conditions are poorly understood. Optical coherence tomography (OCT) is a widely used, fast, and safe examination for providing anterior and posterior segment scans with a high axial and transverse spatial resolution. However, ordinary OCT imaging may be insufficient to image the entire area of the deformed lens due to limited axial range and a relatively low resolution of a posterior lens (9). Therefore, for the diagnosis of congenital lens deformities, a more accurate and efficient biometry measurement device is warranted.

Efforts have been made to image the whole crystalline lens, including the ultra-long scan depth OCT, and complementary metal-oxide-semiconductor-based spectral-domain OCT (10, 11). However, these techniques are generally laboratory-based and still yet to be made accessible for clinical practices. CASIA2 is a new generation swept-source anterior segment optical coherence tomography (SS-ASOCT), offering comprehensive scanning with quantitative measurements of the anterior segment structure. It is able to obtain high-quality images of the posterior lens surface as it measures the tissue with a maximum width of 16 × 16 mm and a maximum depth of 13 mm, by achieving high-resolution images of 10 and 30 μm for the axial and transverse section, respectively (12). Although the CASIA2 scanner enables automatic calculation of anterior and posterior lens curvatures with lens thickness (LT), a few studies have evaluated the lens parameters of congenital lens deformities using CASIA2. Therefore, this study assessed the lens parameters, including MSP, CL, and PL of congenital lens deformities, for investigating the performance of CASIA2 for diagnosing lens diseases.

## Methods

### Patient Eligibility and Ethics Statement

Patients with congenital lens deformities receiving lens surgery at the Eye and ENT Hospital of Fudan University, Shanghai, China were recruited from January 2019 onward. MSP was diagnosed if the entire lens equator was observed under complete pupil dilation (13). The absence of tissues in the equatorial region of the lens was diagnosed as CL (5), whereas a cone-shaped protrusion of the posterior lens surface was defined as PL (8). However, the following exclusion criteria were used for this study: (1) lens deformities secondary to eye trauma or intraocular surgery; (2) lens dislocated into the posterior pole; and (3) other ocular co-morbidities such as retinal detachment, retinal pigmentosa, and end-stage glaucoma. All the procedures were carried out after obtaining ethical approval from the Human Research Ethics Committee of the Eye & ENT Hospital of Fudan University (No. 2020126-1). Informed consent was obtained from all participants and guardians in case the participant was under the age of 18. This study was conducted according to the 1964 Declaration of Helsinki and its later amendments.

### Ophthalmic Examinations

All enrolled patients were examined using a slit-lamp by the same experienced ophthalmologist (YXJ) under complete pupillary dilation. Afterward, the best corrected distance visual acuity (BCVA) was measured by an experienced optometrist. The axial length (AL), corneal keratometry (K), anterior chamber depth (ACD), and LT were obtained using partial coherence interferometry (IOLMaster 700, Carl Zeiss Meditec AG, Jena, Germany) for all the participants. Intraocular pressure (IOP) was measured by a non-contact tonometer (CT-80, Topcon Medical Systems, Oakland, NJ, USA), while fundus examinations were done with OCT (Spectrialis OCT, Heidelberg Engineering, Heidelberg, Germany), OCT (RTVue OCT, Optovue Inc., Freemont, CA, USA), retinal camera (TRC-NW400, Topcon Medical Systems, Oakland, NJ, USA), and ultra-widefield retinal camera (CLARUS 500, Carl Zeiss Meditec AG, Jena, Germany).

The anterior segment was imaged using the swept-source AS-OCT (CASIA2, Tomey Corp, Nagoya, Japan) by the same ophthalmologist (ZXC). In-built software was used to adjust the refraction distortion at the air corneal interface. All the examinations were carried out under complete pupil dilation by the topical administration of tropicamide (Mydrin-P, Santen Pharmaceutical Co., Ltd., Osaka, Japan). The eyelid was kept open with the assistance of the ophthalmologist during the examination. The lens was scanned under the C-scan mode of “Lens Global Scan,” which has taken 128 cross-sectional images by default.

### Genetic Tests and General Examinations

All the participants were screened genetically using panel-based next-generation sequencing (NGS) as reported previously (14). Briefly, genomic DNA was extracted from the peripheral blood samples of the patients. The enriched library was built and sequenced by panel-based NGS generated by Amplicon Gene (Shanghai, China) for the extracted DNA. Sanger sequencing was performed to confirm any candidate mutations of the patients as well as for their family members. All the mutations were classified under the American College of Medical Genetics and genomics guidelines (15).

### Statistical Analysis

All the studied lens biometric parameters, including anterior lens radius (ALR), posterior lens radius (PLR), anteroposterior distance (APD), ACD, and equatorial diameter (Eq Dia) (4), were calculated by the CASIA2 built-in software after the manual alignment of the anterior and posterior surface of the lens in all the acquired sections. For the PL, rear projection length (RPL) and maximum diameter of the lesion (MDL) were measured manually and used as defined in a previous study (16). The MDL was defined as the peak distance between the margins of both the sides of the posterior lesion. Meanwhile, the vertical distance from the center of posterior surface of the lens to the focal protrusion apex was defined as the RPL (Supplementary Figure 1). The mean ALR and PLR were calculated using the built-in algorithm. The calculation of lenticular astigmatism was done based on the steep and flat radius, calculated by the built-in software (Supplementary Figure 2). Considering the left eye of CL, for example, the anterior lens astigmatism (Fa) could be obtained as follows (17):


(1) $$\begin{array}{r} F_{f}=\frac{n_{2}-n_{1}}{R_{f}}=\frac{1.416-1.336}{0.00644}=12.42D \end{array}$$

(2) $$\begin{array}{r} F_{s}=\frac{n_{2}-n_{1}}{R_{s}}=\frac{1.416-1.336}{0.00437}=18.30D \end{array}$$

(3) $$\begin{array}{r} F_{a}=F_{f}-F_{s}=-5.88D \end{array}$$

where *n*_1_ is the refractive index of the aqueous humor, *n*_2_ is the equivalent refractive index of the crystalline lens, *R*_f_ is the flat lens radius, *R*_f_ is the steep lens radius, *F*_f_ is the diopter of the flat meridian, and *F*_s_ is the diopter of the steep meridian. The posterior lens astigmatism was calculated in a similar manner. The APD and Eq Dia were measured manually based on the reconstructed outline of the lens. The obtained continuous data were presented as mean ± SD.

## Results

### Clinical Characteristics

Three patients (*n* = 5 eyes) were enrolled in this study, where one patient was with bilateral MSP (Patient 1), one patient was with bilateral CL (Patient 2), and one patient was with the unilateral PL (Patient 3).

**Figure 1:**
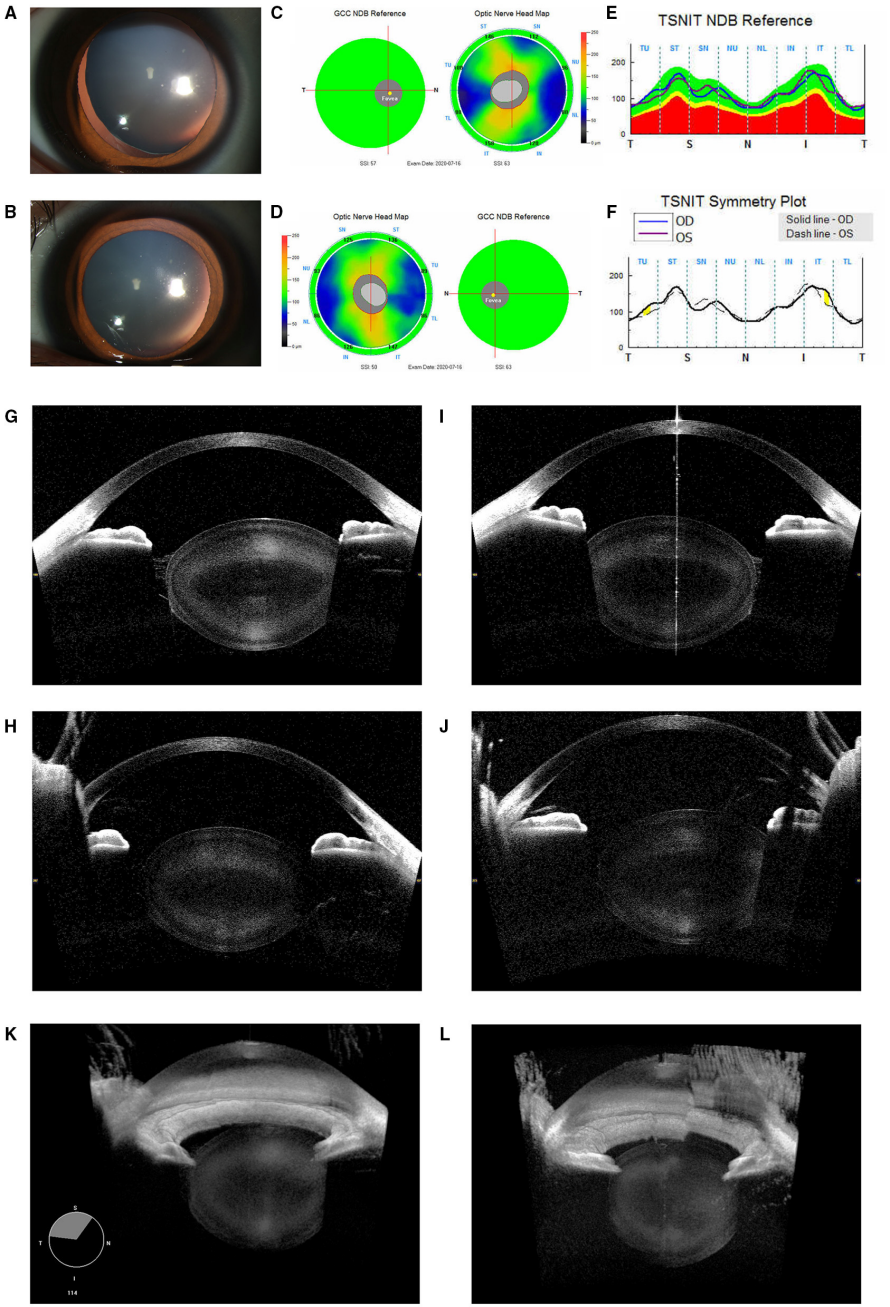
Ocular images of Patient 1 with microspherophakia (MSP). **(A)** A slit-lamp photograph upon mydriasis showed the spherical lens with a mild dislocation in the right eye. **(B)** A slit-lamp photograph upon mydriasis showed the spherical lens with a minimal dislocation in the left eye. **(C–F)** RTVue optical coherence tomography (OCT) of the retinal neural fiber layer showed that no glaucomatous changes were observed in either eyes. **(G)** Horizontal section images of the right eye acquired by the CASIA2 exhibited a small and spherically shaped crystalline lens. **(H)** Similar findings were observed in the vertical section. **(I)** Horizontal section images of the left eye acquired by the CASIA2 exhibited a small and spherically shaped crystalline lens. **(J)** Similar findings were observed in the vertical section. **(K)** Three-dimensional (3D) reconstructed images from the cornea to posterior lens showed a spherical lens in the right eye. **(L)** The left eye had a similar but slightly compromised model due to the eye movement of the patient.

*Patient 1*: A 23-year-old male presented with bilateral decreased vision from 1 year. The BCVA was 0.3 LogMAR with −12.75 DS/−5.50 DC × 120° (OD) and 0.1 LogMAR with −13.00 DS/−5.75 DC × 180° (OS) at the time of examination. Meanwhile, the IOP was 16.8 mmHg (OS) and 23.1 mmHg (OD) under the regular administration of AZARGA (Novartis Lid., Basel, Switzerland). The slit-lamp examination revealed a spherically shaped lens in both the eyes, with a mild and supero-temporal subluxation in the right eye (Figures 1A,B); however, neither of the eyes manifested retinal nerve fiber layer thinning (Figures 1C–F). CASIA2 scanning showed a full range anterior segment with thickened and posteriorly dislocated lenses (Figures 1G–J). These morphological changes were more apparent through three-dimensional (3D) reconstructive images (Figures 1K,L). The physical examination did not reveal any systemic abnormality. The ECG result showed a healthy heart with a normal aortic root. However, the patient's and his mother's genetic testing revealed a heterozygous mutation of the *FBN1* gene (c.3244G > T) in exon26.

**Figure 2:**
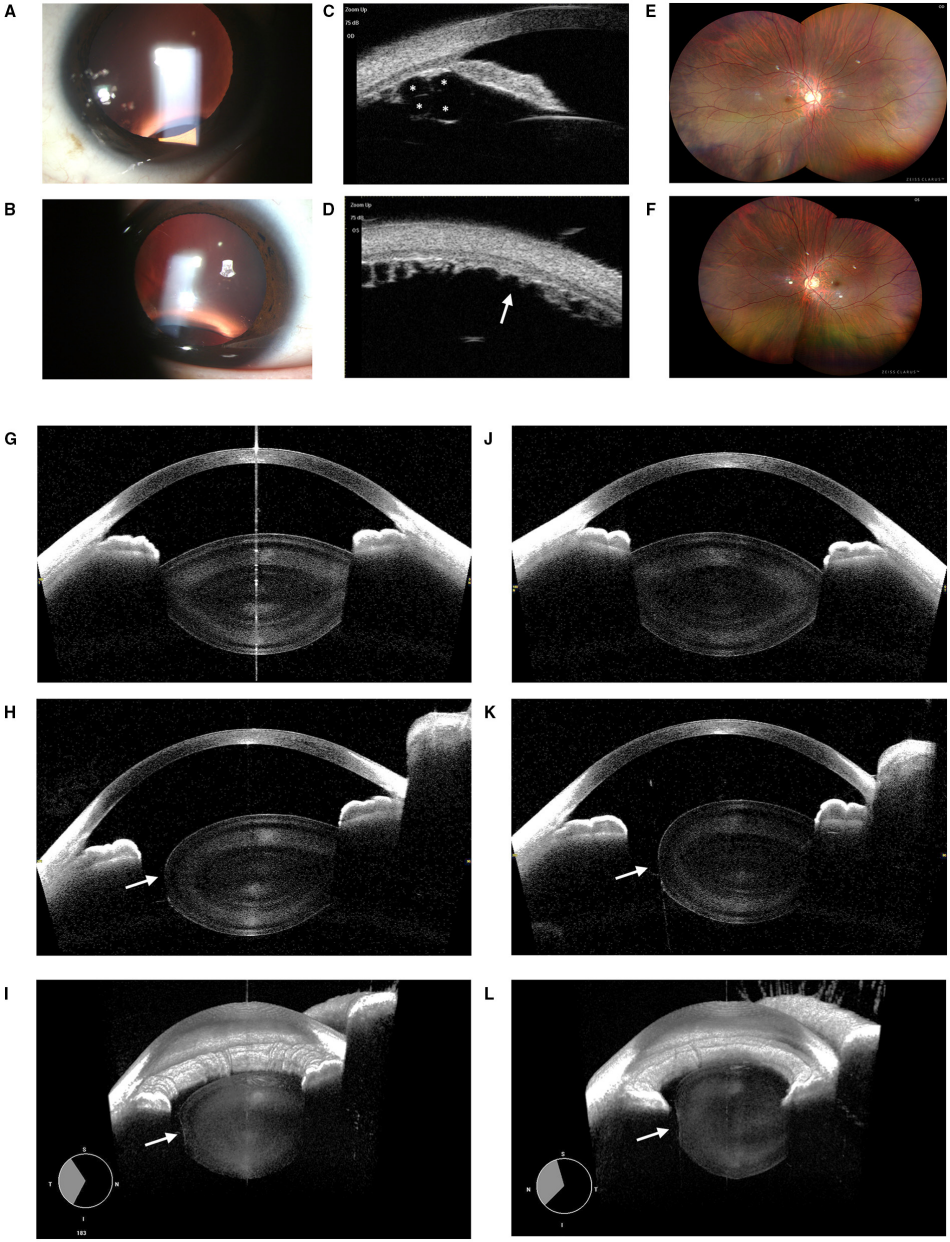
Ocular images of Patient 2 with coloboma lentis (CL). **(A)** A slit-lamp photograph upon mydriasis showed the notching of the lens equator infero-temporally of the right eye. **(B)** A slit-lamp photograph upon mydriasis showed notching of the lens equator inferiorly of the left eye. **(C)** Multiple ciliary body cysts (indicated by “*”) were detected by the ultrasound biomicroscopy. **(D)** The absence of the ciliary body (indicated by “arrow”) was located toward the lens coloboma. **(E)** No retinochoroidal coloboma was observed in the ultra-field retinal photographs of the right eye. **(F)** The left eye showed no retinochoroidal coloboma either. **(G)** Horizontal sections revealed biconvex-shaped but thicker lenses in the right eye. **(H)** The edge of the coloboma was blunt and flattened in the right eye (indicated by an “arrow”). **(J)** Horizontal sections revealed biconvex-shaped but thicker lenses in the left eye. **(K)** The edge of the coloboma was blunt and flattened in the left eye (indicated by an “arrow”). **(I)** 3D reconstructed images of the anterior segment in the right eye. The coloboma was indicated by an “arrow.” **(L)** 3D reconstructed images of the left one.

*Patient 2*: A 19-year-old boy visited the hospital for correcting the refractive error. His BCVAs were 0.0 Log MAR bilaterally at the time of examination. However, high astigmatism was found in both eyes with −12.50 DS/−3.25 DC × 15° (OD) and −13.00 DS/−7.00 × 170° DC (OS). High astigmatism was suspected to be mainly lenticular as corneal astigmatism measured by IOL Master 700 was −1.22D × 13° DC in the right eye and −2.58D × 177° DC in the left eye only. Under mydriasis, no apparent lens dislocation was observed with the equator of the lens being notched in both the eyes, which was suggestive of bilateral lens coloboma at the inferotemporal site (Figures 2A,B). Further, ultrasound biomicroscopy showed multiple ciliary body cysts bilaterally (Figure 2C) and the absent ciliary body toward the coloboma (Figure 2D). Of note, through the ultra-widefield retinal camera, it is revealed that there is no coloboma of either uvea, retina, or optic nerve (Figures 2E,F). The CASIA2 scanner showed wide coverage of the lens (128 sections in total), therefore the edge of the coloboma could be clearly observed. The observed coloboma ranged from 250° to 311° in the right eye, whereas from 222° to 308° in the left eye as measured by CASIA2. Horizontal sections revealed biconvex-shaped but thicker lenses in both the eyes (Figures 2G,J), while the edge of the coloboma was blunt and similar to that of MSP (Figures 2H,K). Moreover, similar changes were discovered by 3D reconstructive imaging (Figures 2I,L). On physical examination except for pigeon breast, no positive evidence of any systemic disease was found. Also, no abnormality was detected by ECG. The genetic test revealed a heterozygous mutation in the *FBN1* gene (c.290C > G), inherited from his mother.

**Figure 3:**
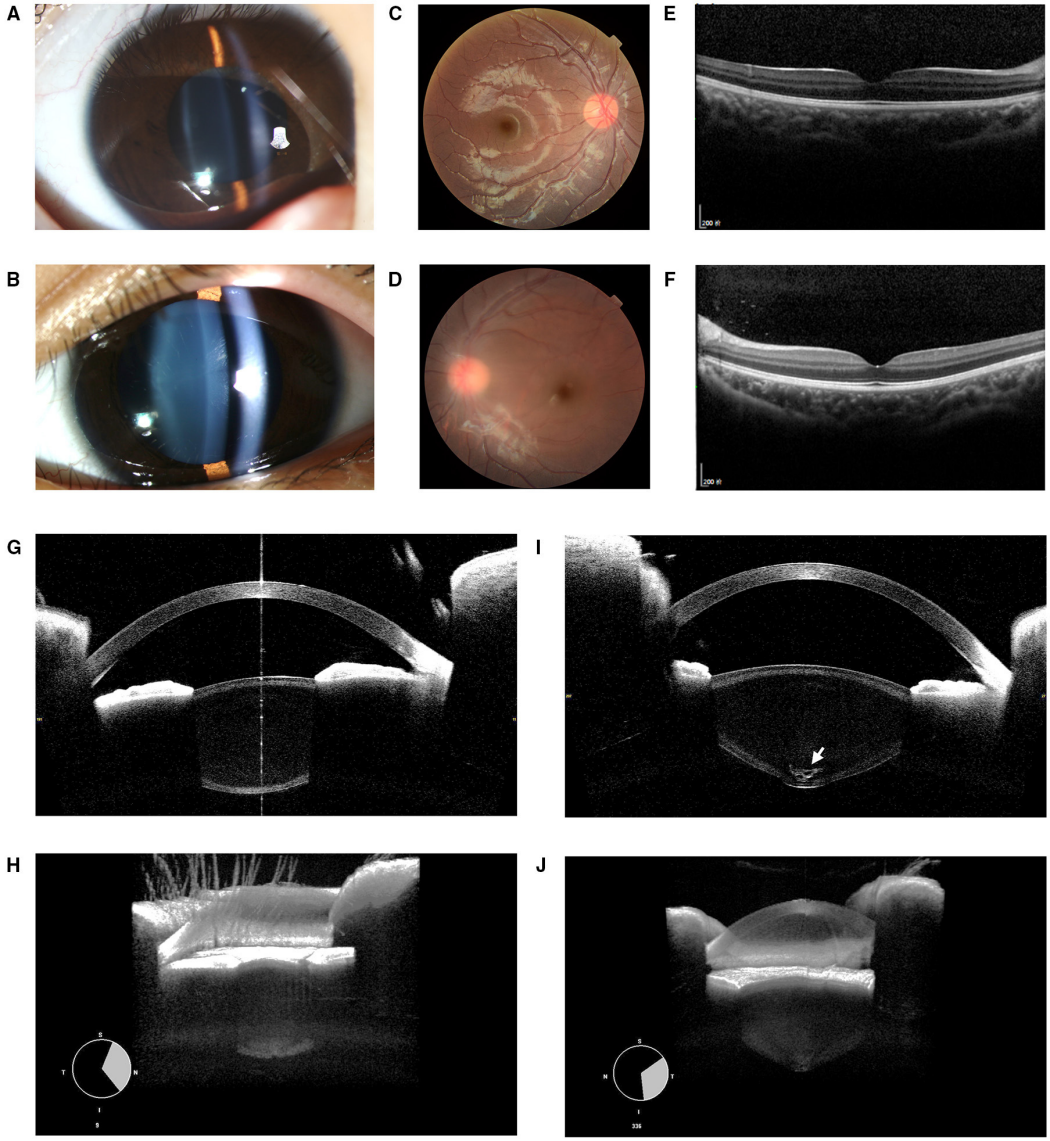
Ocular images of Patient 3 with posterior lenticonus (PL). **(A)** A slit-lamp photograph showed a normal lens in the right eye. **(B)** The light beam showed a cone-shaped protrusion of the posterior lens surface in the left eye with no apparent posterior polar cataract. **(C–F)** Abnormities of fundi were found in either eyes. **(G)** Normal lens image obtained by CASIA2. **(I)** The PL associated with posterior polar cataract (indicated by an “arrow”) of OS was found. **(H)** 3D reconstructed images showed the normal anterior segment in the right eye. **(J)** A cone-shaped protrusion of the posterior lens was reconstructed in the left eye.

*Patient 3*: A 6-year-old girl presented with poor vision in her left eye since childhood. The BCVA was 0.0 and 0.5 for the right and left eye at the time of examination. The manifest refraction was +0.25 DS in the right and +0.25 DS/−1.75 DC × 150° in the left eye, indicating that the amblyopia in the left eye was probably due to astigmatism. Upon the slit-lamp biomicroscopic examination, a clear but conical lens in the left eye as compared to the right eye was observed (Figures 3A,B), whereas the fundus examinations revealed no obvious abnormality (Figures 3C–F). High-definition images of the anterior and posterior surface of lenses were obtained by CASIA2, exhibiting a very typical PL with a tiny posterior cataract in OS (Figures 3I,J) with the normal lens in OD (Figures 3G,H). The systematic examinations and genetic testing results were unremarkable.

### Ocular and Lens Biometry

Both ocular and lens biometrics measured in this study are summarized in Table 1, together with a comparative analysis of other published relevant studies (16, 18–23). The ALR and PLR were smaller than the normative values for the eyes of MSP and CL; however, a decrease in the ALR was more prominent than that of PLR. For the patient with CL, the mean ALR, PLR, and APD were slightly greater than that of MSP. The lenticular astigmatism was also prominent in the eyes of CL. The steep and flat lens radius measured by CASIA2 demonstrated −1.87DC × 13° astigmatism in the anterior lens and −1.02DC × 15° astigmatism in the posterior lens for the right eye, while −5.88DC × 171° astigmatism in the anterior lens and −4.00DC × 153° astigmatism in the posterior lens for the left eye were observed. The PL eyes had extremely high posterior surface curvatures. Other parameters of the measured eyes were within the normal range. The Eq Dia was smaller in MSP eyes and was comparable to the previously reported studies. The decentration amount (0.13 mm in the right eye and 0.68 mm in the left eye) and tilting angle (8.3° in the right eye and 4.1° in the left eye) of CL eyes as calculated by CASIA2 were in the normal range of 0.41 ± 0.25 mm and 4.8° ± 3.48°, respectively, indicating no lens subluxation.

**Table 1:** Ocular and lens biometric parameters in this study and existing literature.

**References**	**Condition**	**Age/y**	**OD/ OS**	**Ocular biometry /mm**			**Lens biometry/mm**											
				**AL** **/mm**	**K**_**m**_ **/D**	**LT** **/mm**	**Device**	**ALR/mm**			**PLR/mm**			**APD^#^** **/mm**	**ACD^#^ /mm**	**Eq Dia^#^** **/mm**	**MDL^#^ /mm**	**RPL^#^** **/mm**
								**ALR**_**s**_	**ALR**_**f**_	**ALR**_**m**_	**PLR**_**s**_	**PLR**_**f**_	**PLR**_**m**_					
Current study	MSP	22	OD	26.66	42.00	5.27	SS–OCT	4.60	5.42	5.01	4.03	4.12	4.08	5.39	3.00	7.42	–	–
			OS	26.84	42.16	5.35		4.97	5.32	5.14	4.03	4.25	4.14	5.49	3.37	7.22	–	–
	CL	19	OD	26.16	42.37	4.28		5.98	6.95	6.46	4.38	4.64	4.51	4.45	2.58	8.38	–	–
			OS	26.14	42.44	4.37		4.37	6.44	5.41	3.89	4.83	4.36	4.58	2.42	8.00	–	–
	PL	4	OD	20.89	46.31	3.88		8.91	9.15	9.03	4.75	5.64	5.20	3.86	2.64	9.07	–	–
			OS	21.42	46.62	3.99		8.94	9.45	9.19	1.58	1.76	1.67	4.07	2.42	8.96	1.91	0.28
Chan et al. (18)	MSP	9	OD	21.68	–	4.77	Scheimpflug camera	–	–	6.2	–	–	6.3	–	1.57^#^	–	–	–
			OS	21.79	–	4.89		–	–	6.3	–	–	5.6	–	1.37^#^	–	–	–
Burakgazi et al. (19)	MSP	26	OD	23.3	–		NA	–	–	–	–	–	–	–	2.63	7.5	–	–
			OS	25.0	–			–	–	–	–	–	–	–	2.40	8.0	–	–
Lim et al. (20)	MSP	37	OD	22.03	–	–	Caliper	–	–	–	–	–	–	7.0	–	6.75		
			OS	21.74	–	–		–	–	–	–	–	–	6.75	–	6.50		
Shakrawal et al. (21)	MSP	13	OD	22.87	–	4.06	UBM	–	–	–	–	–	–	–	0.97	6.56	–	–
			OS	23.01		4.09		–	–	–	–	–	–	–	0.55	6.87		
Liu et al. (22)	MSP	7	OD	22.97	–	5.07	Color ultrasound	–	–	–	–	–	–	–	3.53	6.2	–	–
			OS	22.61	–	5.05		–	–	–	–	–	–	–	3.40	6.3		
Chen et al. (16)	PL	31.5 ± 34.8		21.04 ± 1.17	–	–	Scheimpflug camera	–	–	–	–	–	–	–	–	–	–	0.80 ± 0.43
Takuhei et al. (23)	Health control	26.6 ± 4.3	Random one	25.3 ± 1.4	–	3.7 ± 0.2	SS–OCT	–	–	11.5 ± 1.3	–	–	6.1 ± 0.4	–	–	–	–	–
		47.2 ± 6.5		24.2 ± 1.5	–	4.2 ± 0.3			–	9.5 ± 1.3	–	–	5.8 ± 0.3	–	–	–	–	–

*ACD, anterior chamber depth; AL, axial length; ALR, anterior lens radius; APD, anteroposterior distance; CL, coloboma lentis; Eq Dia, equatorial diameter; K, keratometry; MDL, maximum diameter of the lesion; MSP, microspherophakia; PL, posterior lenticonus; RPL, rear projection length; PLR, posterior lens radius; UBM, ultrasound biomicroscopy; m, mean; f, flat; s, steep; NA, not applicable*.

# * Measured in the horizontal section*.

**Figure 4:**
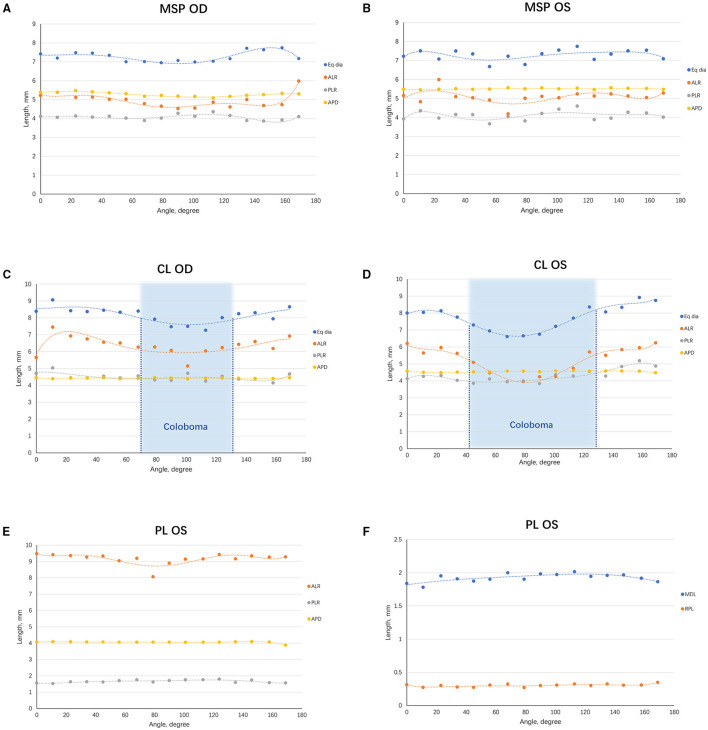
Scatterplots demonstrating the trend of lens biometric parameters from various angles. **(A)** The lens biometric parameters of the right eye of MSP at different angles were plotted and fitted in curves. **(B)** A similar graph was made on the left one. **(C)** The lens biometric parameters of the right eye of CL at different angles were plotted and fitted in curves. The coloboma ranged from 250° to 311° (indicated in the blue area). **(D)** A similar graph was made on the left one. A larger range of coloboma was observed in the left eye (222° to 308°, indicated in the blue area). **(E)** The ALR, PLR, and APD of the eye with PL. **(F)** The MDL and RPL of the eye with PL. ACD, anterior chamber depth; ALR, anterior lens radius; APD, anteroposterior distance; CL, coloboma lentis; Eq Dia, equatorial diameter; MDL, maximum diameter of the lesion; MSP, microspherophakia; PL, posterior lenticonus; RPL, rear projection length; PLR, posterior lens radius.

The lens biometrics at all angles were analyzed using the C-scan mode of CASIA2, which offers an opportunity to visualize the globular lens. The ALR, PLR, Eq Dia, and APD were similar in MSP eyes at all the angles, suggestive of homogeneous biometry in lenses of MSP (Figures 4A,B). For CL eyes, the ALP and Eq Dia decreased sharply at the coloboma region, whereas the PLR and APD were barely affected, indicating that the anterior surface of the lens was more susceptible to the coloboma (Figures 4C,D). Besides, the ALP, PLR, Eq Dia, MDL, and RPL were similar at different angles in the PL eye (Figures 4E,F).

## Discussion

As a significant component of the ocular refractive media, the lens has the function of transmitting light and focusing it on the retina. A pair of the normal crystalline lens contributes to a clear binocular vision, a prior condition for human visual development (24). With the advancements in ocular imaging, the biometry of the cornea, anterior chamber, and retina has been extensively characterized. However, when it comes to the lens biometric parameters, still many unexplored domains exist, especially for congenital deformities (25). In the current study, we used CASIA2, a novel swept-source anterior segment OCT, for investigating the morphology as well as the biometric parameters of MSP, CL, and PL in patients. High spatial resolution, deep penetrating, fast scanning, and non-invasive nature of this technique are of immense help for clinicians for arriving at an accurate diagnosis and exploring the pathogenesis of congenital lens deformities.

A precise and accurate measurement of lens parameters is very crucial for early diagnosis and a timely surgical intervention (8, 26). Although MSP is a rare congenital ocular disorder, it is known for its complications such as lens dislocation, high lenticular myopia, with secondary glaucoma (18). Still, an early diagnosis of MSP is a very cumbersome task. The crystalline lens is small and spherical (27), but the estimation of the lens size by the slit-lamp examination may be limited by either limiting pupil dilation associated with Marfan syndrome or poor cooperation of young patients (28). In the current study, due to the mild subluxation, the equators could not be fully visualized. The increased lens curvature, APD, and decreased Eq Dia assessed by CASIA2, facilitated the early diagnosis of MSP in this case. Meanwhile, its non-contact nature and fast capturing speed (0.016 s per cross-section) not only promoted the compliance of the young children but also reduced the risk of associated contact infection, especially during the pandemic of COVID-19. Of note, the eyes of Marfan syndrome often had vitreous liquefaction at the base, and thus the lens of MSP may dislocate posteriorly and eventually into the posterior pole (29). In the current case of MSP, the distance between the anterior surface of the cornea and the posterior surface of the lens was 8.95 and 9.40 mm in the right and left eye, which exceeded the scanning depth of spectral-domain OCT devices. Therefore, by using CASIA2, we could frame all the pellucid structures of the anterior segment in a single image.

The altered lens biometrics can shed light into the potential pathophysiology of lens deformities. The morphology of MSP, CL, and PL has been extensively depicted by the histological studies of the extracted lens or cadaver eyes, yet dehydration and slice making of the lens are important concerns for such examinations (8, 20, 30). Although ultrasound biomicroscopy has the advantage of penetrating the iris and ciliary body, it might induce morphological changes in the eyeball due to its contact nature (31). Therefore, the application of CASIA2 makes it possible to examine and study the lens *in vivo*. The CL is a rare lens disease, possessing concavity of the lens in the equator due to regional maldeveloped zonules (32). In addition to limited slit-lamp photos in the published case reports, the morphological features of CL largely remain elusive. In the current study, we acquired a 360° view of the lenses of our patient using the CASIA2. During the examination, a continuous and blunt-edged margin of the coloboma was identified for the first time. The edge of CL contained a certain similarity with MSP, suggestive of a severe loss of zonule strength at the site of coloboma. We have also seen a significant decrease of ALR at the notch site, while the PLR and APD were independent of the coloboma. Thus, our findings further indicate that the lens biometrics of an anterior surface are more susceptible to the loss of zonules in comparison with a posterior surface. It is also noteworthy that the ALR was more prone to the accommodation in the healthy lens (23). Collectively, these findings indicate a greater strength imposed by the anterior tines of equatorial zonules than that of the posterior ones. Moreover, “true” coloboma is caused by a defective closure of the embryonal fissure, involving mutilation of the optic disc, retina, choroid, iris, ciliary body, and lens (33). Though the ocular multisegment coloboma has been reported in a Marfan syndrome patient (13). In terms of the patient in our study, the patient's lens coloboma might not be a “true” coloboma as the iris, choroid, and retina were equipped. The “isolated” CL is largely attributed to the maldeveloped ciliary body, as revealed by the ultrasound biomicroscopy. Subsequently, the absence of the force applied by the zonules leads to the notch of the lens. The residual zonules probably carry subclinical defects, inferring from a slightly increased APD. The coexistence of maldeveloped ciliary body and defective zonules makes sense as both of them develop from mesoderm, which is defective in the setting of Marfan syndrome and related fibrillinopathy (34).

For most optical techniques, the posterior surface of the lens imaging is challenging due to the overlap of the real and mirror images from the cornea and lens, where the refraction distorts the images. However, the visualization of the posterior surface is important for diagnosing and understanding the opacity and deformities of the posterior lens. The PL is a rare ectasia of the posterior capsule of the lens, with the clinical appearance of localized, oval, and well-circumscribed protrusion of the posterior lens surface (35). The widely accepted theory elucidating its pathogenesis, states that the PL develops by the herniation of posterior capsule and cortical lens fibers into the vitreous at a region of posterior capsule weakness under increasing intralenticular pressure (8). However, the detection of PL in a clear lens often requires an experienced and a careful observation during the slit-lamp examination. Many studies have used the Scheimpflug imaging system to diagnose PL (16). However, its visible light source limits the penetration and also the resolution of the lens images. A study has reported that the posterior lens could be measured by Scheimpflug imaging, but only in 6% of patients with a mild refractive error or cataract (36). In our study, using CASIA2, we could easily obtain a global view of PL and therefore detected even the subtle posterior polar cataract. Our data revealed that CASIA2 can serve as an ideal tool for an early and accurate diagnosis of PL, leading to early surgical intervention with a better visual prognosis.

A major limitation of the current study was the limited number of enrolled eyes. However, included conditions were extremely rare in clinical practices. Therefore, even in the small number of eyes, we had the opportunity to analyze each deformed lens in detail. Meanwhile, as pupil dilatation is a pre-requisite for lens imaging, the lens biometrics and conclusions of this study are only applicable for the mydriasis state, which might be slightly different from the normal pupil state (23). All in all, high-resolution OCT sections, 3D reconstructed images, and biometrics parameters of this study would provide meaningful information for both ophthalmologists and researchers.

In conclusion, the advancements in SS-ASOCT offer a promising tool for *in vivo* assessment of lens biometry. The CASIA2-assisted observation in cases of MSP, CL, and PL, and the build-in-software facilitates measuring of the lens biometrics after a proper alignment. Therefore, the CASIA2 could be of great value in assisting ophthalmologists to reach an early diagnosis and further providing illuminating readings for the lens pathophysiology studies.

## Data Availability Statement

The original contributions presented in the study are included in the article/Supplementary Materials, further inquiries can be directed to the corresponding author.

## Ethics Statement

The studies involving human participants were reviewed and approved by Human Research Ethics Committee of the Eye and ENT Hospital of Fudan University. Written informed consent to participate in this study was provided by the participants' legal guardian/next of kin. Written informed consent was obtained from the individual(s), and minor(s)' legal guardian/next of kin, for the publication of any potentially identifiable images or data included in this article.

## Author Contributions

Z-xC conceived and designed the experiments. Z-xC and W-NJ collected the clinical samples. Z-xC and Y-XJ performed clinical examinations of patients. Z-xC, W-NJ, and Y-XJ drafted and revised the manuscript. All authors read and approved the manuscript.

## Funding

This study was supported by the National Key R&D Program (Grant No. 2018YFC0116004), the National Natural Science Foundation of China (Grant Nos. 81770908 and 82070943), and the Shanghai Science and Technology Commission (Scientific Innovation Action Plan, Grant No. 18411965200).

## Conflict of Interest

The authors declare that the research was conducted in the absence of any commercial or financial relationships that could be construed as a potential conflict of interest.

## Publisher's Note

All claims expressed in this article are solely those of the authors and do not necessarily represent those of their affiliated organizations, or those of the publisher, the editors and the reviewers. Any product that may be evaluated in this article, or claim that may be made by its manufacturer, is not guaranteed or endorsed by the publisher.
