# Supplementary material for: Lens Biometry in Congenital Lens Deformities: A Swept-Source Anterior Segment OCT Analysis
Source: Front Med (Lausanne). 2021 Dec 20;8:774640. doi: 10.3389/fmed.2021.774640 (PMC8720848; doi:10.3389/fmed.2021.774640)
Supplement: Supplementary file 1 [file Data_Sheet_1.docx]

Supplementary Material

# Supplementary Figures


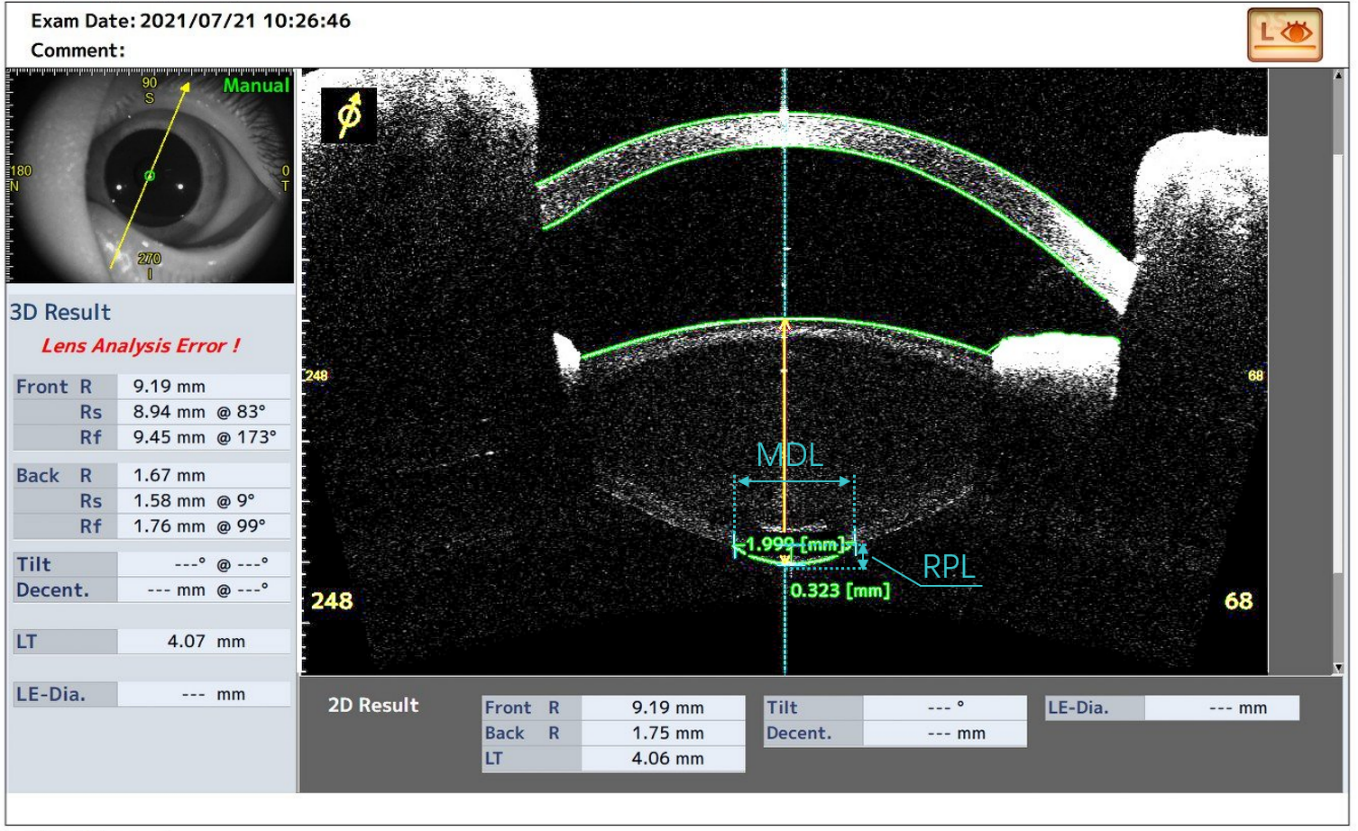


**Supplementary Figure S1.** Maximum diameter of the lesion (MDL) was defined as the largest distance between the margins of both sides of the posterior lesion in cross-sectional images from different directions. Rear projection length (RPL) was defined as the perpendicular distance from the center of the lens posterior surface to the focal protrusion apex.


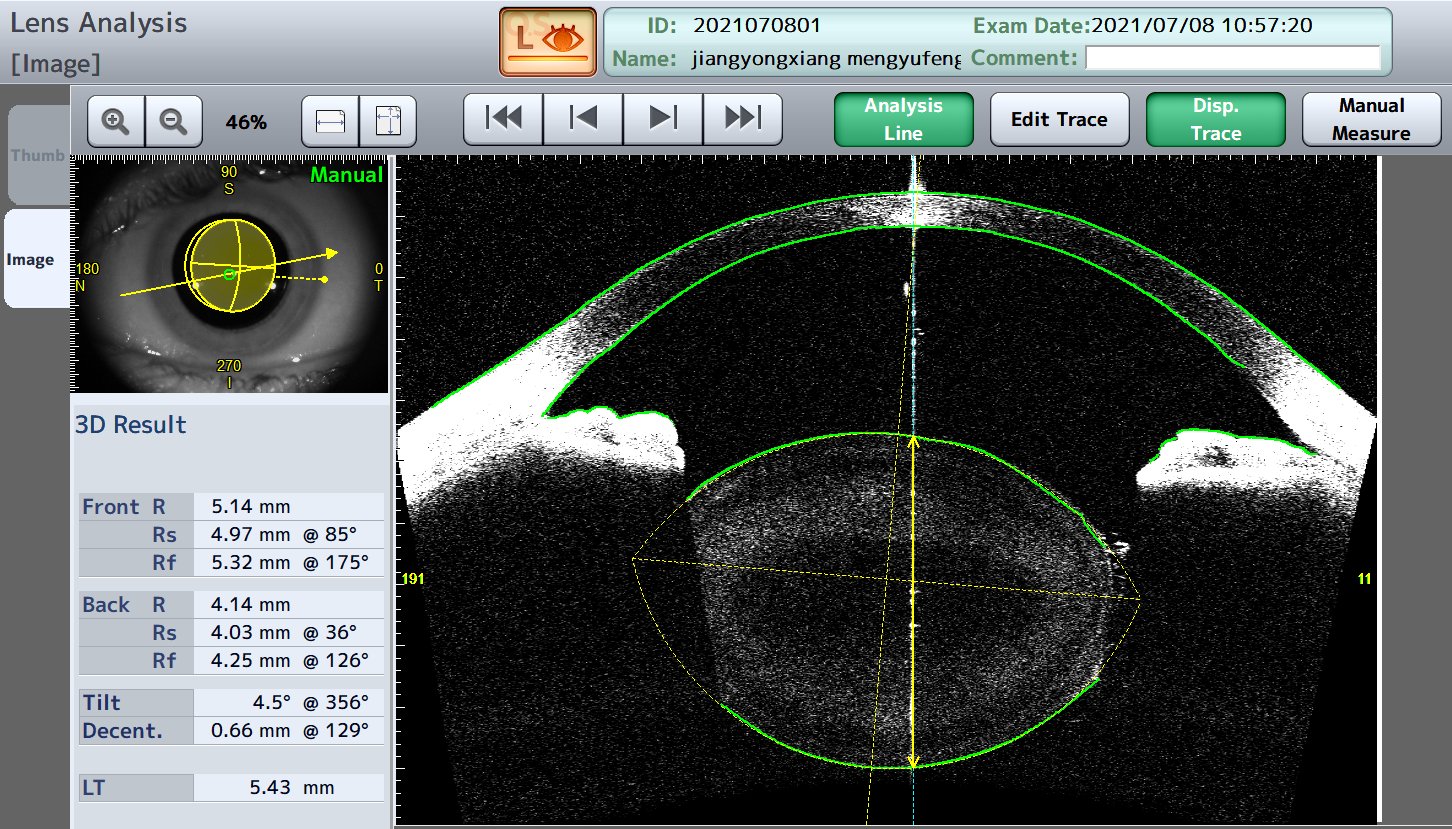


**Supplementary Figure S2.** In this CASIA2 screenshot of microspherophakia (MSP), anterior and posterior surface of the lens were delineated manually followed by automatic alignment, then the steep lens radius (R_s_) and flat lens radius (R_f_) could be calculated by the built-in software on the left column.
